# Identification of doping suspicions through artificial intelligence-powered analysis on athlete’s performance passport in female weightlifting

**DOI:** 10.3389/fphys.2024.1344340

**Published:** 2024-06-13

**Authors:** Hyunji Ryoo, Samuel Cho, Taehan Oh, YuSik Kim, Sang-Hoon Suh

**Affiliations:** ^1^ Department of Physical Education, Yonsei University Graduate School, Seoul, Republic of Korea; ^2^ Independent Researcher, Seoul, Republic of Korea; ^3^ Severance Institute for Vascular and Metabolic Research, Yonsei University College of Medicine, Seoul, Republic of Korea; ^4^ Department of Physical Education, College of Educational Sciences, Yonsei University, Seoul, Republic of Korea

**Keywords:** athlete’s performance passport (APP), doping, anti-doping, artificial intelligence (AI), female weightlifting

## Abstract

**Introduction:**

Doping remains a persistent concern in sports, compromising fair competition. The Athlete Biological Passport (ABP) has been a standard anti-doping measure, but confounding factors challenge its effectiveness. Our study introduces an artificial intelligence-driven approach for identifying potential doping suspicious, utilizing the Athlete’s Performance Passport (APP), which integrates both demographic profiles and performance data, among elite female weightlifters.

**Methods:**

Analyzing publicly available performance data in female weightlifting from 1998 to 2020, along with demographic information, encompassing 17,058 entities, we categorized weightlifters by age, body weight (BW) class, and performance levels. Documented anti-doping rule violations (ADRVs) cases were also retained. We employed AI-powered algorithms, including XGBoost, Multilayer Perceptron (MLP), and an Ensemble model, which integrates XGBoost and MLP, to identify doping suspicions based on the dataset we obtained.

**Results:**

Our findings suggest a potential doping inclination in female weightlifters in their mid-twenties, and the sanctioned prevalence was the highest in the top 1% performance level and then decreased thereafter. Performance profiles and sanction trends across age groups and BW classes reveal consistently superior performances in sanctioned cases. The Ensemble model showcased impressive predictive performance, achieving a 53.8% prediction rate among the weightlifters sanctioned in the 2008, 2012, and 2016 Olympics. This demonstrated the practical application of the Athlete’s Performance Passport (APP) in identifying potential doping suspicions.

**Discussion:**

Our study pioneers an AI-driven APP approach in anti-doping, offering a proactive and efficient methodology. The APP, coupled with advanced AI algorithms, holds promise in revolutionizing the efficiency and objectivity of doping tests, providing a novel avenue for enhancing anti-doping measures in elite female weightlifting and potentially extending to diverse sports. We also address the limitation of a constrained set of APPs, advocating for the development of a more accessible and enriched APP system for robust anti-doping practices.

## Introduction

The utilization of banned substances for performance enhancement, commonly referred to as doping, has been a persistent concern since its initial detection in the 1960s, as it compromises the fundamental tenets of fair competition within sporting events (Connor et al., 2013). Over the years, various measures and strategies have been introduced to address the issue and rectify the lack of awareness regarding anti-doping regulations while fortifying doping control (Lauritzen and Holden, 2023). One prominent approach, the Athlete Biological Passport (ABP), involves the indirect detection of biomarkers derived from biological samples obtained from athletes (Saugy et al., 2014). Despite the ABP’s emergence as a standard test for identifying anti-doping rule violations (ADRVs), there persists a need for advancing methodologies in the realm of anti-doping practices, primarily due to the influence of confounding factors on the current variables used in ABP assessments. These factors encompass the use of prescribed medications for unrelated health conditions, individual hematological and endogenous variations (Zorzoli et al., 2014). Furthermore, the effectiveness of ABP may be hindered by the exploitation of the drug detection window and the delayed yet sustained performance enhancement derived from doping practices (Puchowicz et al., 2018). In fact, the actual prevalence of doping considerably exceeds the estimated prevalence in the realm of adult elite sports (de Hon et al., 2015).

Given that the primary objective of doping among athletes revolves around the enhancement of athletic performance, the Athlete’s Performance Passport (APP), which encompasses demographic profiles and performance data, presents an opportunity to identify unusual improvements and/or sustained high-performance records. Previous observations, linking performance changes with trends in doping practices (Schumacher and Pottgiesser, 2009; Iljukov and Schumacher, 2017; Iljukov et al., 2020), support that APP can be effectively leveraged in the efforts to detect ADRVs. Notably, substantial performance improvements were observed in middle- and long-distance runners and professional cyclists after the introduction of commercially available recombinant human erythropoietin, followed by a marked decline in performance as anti-doping measures were reinforced (Schumacher and Pottgiesser, 2009; Perneger, 2010). The utilization of APPs in anti-doping practice, incorporating statistical analyses and AI algorithms, has been recently introduced as well. Based on the performance results in track and field, previous studies conducted statistical analysis on the variations in an athlete’s standardized performance throughout their career, with a focus on distinguishing between clean and doped athletes (Hopker et al., 2020; Hopker et al., 2023). Through the analyses, they introduced the statistical model capable of identifying the differences between these two groups and determining the volatility in performance over an athlete’s career (Hopker et al., 2020), as well as the model capable of identifying unusual improvement in performance compared to their age-matched peers (Hopker et al., 2023). Both of these studies demonstrated the potential for modeling athlete performance data in the risk stratification based on athletes’ likelihood of doping. In this context, the APP may serve as an indirect marker or a means to establish criteria for recognizing potential doping suspicions. It is worth noting that the identification of potential doping suspicions through the APP may be less conspicuous in sports where competition settings lack standardization and performance outcomes are expressed in discrete variables. Nevertheless, the utility of the APP in testing for ADRVs appears evident in sports where an athlete’s physical capacity is the primary determinant of performance within standardized settings, such as track and field, weightlifting, cycling, and swimming (Puchowicz et al., 2018). In this regard, the development of accurate models for targeting suspicious athletes based on APP can provide secondary evidence for establishing criteria to target and test individuals with doping suspicions. Despite prior studies introducing the application of the APP in anti-doping measures, its implementation remains in the nascent stage, warranting further scholarly investigation.

Widespread doping practices in elite weightlifting have presented a significant and ongoing. A striking example of this problem is evident in the fact that among the 515 participants in the Beijing 2008 and London 2012 Olympic Games, 30 weightlifters were subjected to the retroactive revocation of medals, prompting the International Olympic Committee (IOC) to require the International Weightlifting Federation (IWF) to devise a comprehensive anti-doping strategy to avoid exclusion from the Paris 2024 Olympic Games (Kolliari-Turner et al., 2021). Despite positive steps taken by the IWF to address this issue, numerous sanctioned cases continue to be reported by various organizations. This raises questions about whether sufficient measures against ADRVs have been implemented, especially in the countries with a longstanding history of doping. Weightlifting is a sport that places premium on both speed and strength (Morris et al., 2022). Notably, the performance of the weightlifters is significantly influenced by athletes’ body weights (BW) (Ryoo et al., 2022). Age has been identified as another pivotal factor in weightlifting performance, as biological aging has been shown to be associated with performance decline after an athlete’s mid-twenties (Huebner and Perperoglou, 2019). Moreover, weightlifting is a sport in which performance outcomes are precisely quantified in discrete values of the total weight lifted in kilograms (*kg*), and a significant number of ADRVs have been documented (Lauritzen and Holden, 2023). These factors collectively highlight the suitability of weightlifting for conducting research utilizing the APPs in anti-doping practice. However, the extensive history of doping in elite weightlifting may pose significant challenges for the effectiveness of APP in anti-doping practice, especially if doping practices have been initiated before the introduction of detection methods for certain types of substances and their metabolites or before athletes reach high performance levels as the identification of doping suspicious based on APPs primarily relies on unusual deviations from an athlete’s established physiological parameters. In addition, the detection of the unusual deviations from the estimated performance ranges in the athletes’ age groups and BW classes may underestimate the efforts of the athletes during their training. However, the observed decline in performance results in elite weightlifting during 2016–2022 compared 2009–2015, which may partly be attributed to the implementation of new methods to detect long-term metabolites of certain banned substances (Bezuglov et al., 2024)*,* emphasizes the importance of systematical monitoring of the performance data and sanction status over time, as trends in the performance results of sanctioned and non-sanctioned athletes may differ from those of the past. A comparative analysis of athletes’ performance data within their respective BW classes, individuals’ BW, history of sanctions, and ages can establish a reliable basis for identifying potential doping suspicions in weightlifting.

The application and integration of artificial intelligence (AI)-powered algorithms have the potential to significantly enhance not only the efficiency of anti-doping practice (Rodriguez Duque et al., 2023) but also the fairness of competitions. AI’s ability to analyze datasets, such as APP, may efficiently enables the identification of anomalies and irregularities; this, *in turn*, allows anti-doping organizations to allocate their resources more effectively and prioritize testing based on data-driven insights, strengthening the integrity of competitive sports.

In this study, we conducted a comprehensive analysis of the athletic performances of elite female weightlifters, categorizing them based on their sanction status, across a range of performance predictors. Our primary objective was to assess the potential utility of APP in identifying athletes with suspected doping involvement. Furthermore, we undertook the development and validation of an innovative APP-based prediction model for potential doping suspicion, utilizing machine learning techniques.

## Materials and methods

### Data acquisition and processing

The performance data of female weightlifters along with their demographical data were analyzed to evaluate the applicability of the APP in the identification of potential doping suspicions. All data used in this study were sourced from publicly available records on the International Weightlifting Federation (IWF)’s official website (www.iwf.net) and were granted an exemption by the Institutional Review Board of Yonsei University. The dataset encompassed demographic data and performance outcomes of women weightlifters across all competitions organized by the IWF from 1998 to 2020. Demographic data included genders, ages, BW, and doping history; performance outcomes included the total weight lifted (*in* kilograms*, Kg*), which constituted the sum of the best snatch and clean and jerk results, each comprising three attempts. A total of 19,591 records were acquired, 2,533 were removed if the entity contained no performance records, age, or BW, yielding a total of 17,058 records with 15,404 belongs to athletes with no history of being sanctioned (the not-sanctioned group) and 1,654 belonging to athletes with a history of being sanctioned (the sanctioned group). Athletes with sanctions for ADRVs were identified through the IWF sanction list, which designated athletes as ‘DSQ’ for testing positive for prohibited substances in specific events. The criteria for sanctioning aligned with the World Anti-Doping Code, specifically Article 2.1, indicating the “presence of a prohibited substance or its metabolites or markers in an athlete’s sample,” and Article 2.2, defining “use or attempted use by an athlete of a prohibited substance or a prohibited method.” All female weightlifters in the sanctions list were found to have violated either Article 2.1 or 2.2.

For benchmark analysis across various demographic and performance parameters, the dataset systematically categorized the weightlifters into three parameters: age groups, body weight classes, and performance levels. Age was classified into seven groups: under 15, 15–19, 20–24, 25–29, 30–34, 35–39, and 40 and more; body weight in *kg* was classified into seven classes: 49, 55, 59, 64, 76, 87, and +87; and performance level was classified into eight groups: top 1%, top 1%–5%, top 5%–10%, top 10%–25%, top 25%–50%, top 50%–75%, top 75%–90%, top 90%–100%. To facilitate a comprehensive analysis and interpretation, performance outcomes across age groups and BW classes were graphically plotted. Sanction status in performance levels across age and BW class categories were summarized in tables. Python programming language (Ver. 3.9.6; Python Software Foundation, Beaverton, OR, USA) was utilized for all data processing.

### Machine learning approaches for detection of doping suspicions in weightlifting

To ensure the relevance and completeness of our dataset, we identified five key features: age, body weight, snatch record, jerk record, and individual’s belonging body weight class. The dataset was filtered to include senior weightlifters (≥15 years of age) in seven body weight classes described above, with weight class encoded into an ordinal variable ranging from 0 to 6, representing the lightest to the heaviest class. Entities (rows) with a non-zero total (*snatch* + *jerk*) and valid results from all three attempts for both lifts were retained while entities with BW less than 65 kg or higher than 125 kg were removed, resulting in a training set comprising 8,948 entities. The exclusion of entities under 65 kg, a common weight for female weightlifters, aimed to prevent potential hindrance to AI model performance, possibly due to unpredictable patterns in the dataset. For evaluating the performance of the AI-powered models, the outcomes from the dataset of the participants of 2008 Beijing Olympics, 2012 London Olympics, and 2016 Rio de Janeiro Olympics were described. This data includes age, snatch record, jerk records, individual BW and her belonging BW class, which was encoded into ordinal variables identical to those in the training dataset.

To develop AI-powered methodology for the identification of doping suspicions in female weightlifting based on the APPs, we implemented XGBoost and Multilayer Perceptron models to optimize predictive power and the Ensemble model combining the strengths of XGBoost and Multilayer Perceptron (MLP) to further maximize predictive performance. The models were developed based on the analyses using R (Ver. 4.3.1), employing the XGBoost package (Version 1.7.5.1) for gradient boosting and the Neuralnet package (Version 1.44.2) for neural network implementation. XGBoost, a decision tree-based ensemble model, was chosen for its proven efficacy in handling tabular data and its ability to sequentially enhance weak classifier models (Sacks et al., 2018). We fine-tuned our AI model by systematically exploring different configurations through a process known as grid search, which involved optimizing crucial hyper-parameters, including the *max_depth*, *gamma*, *colsample_bytree*, and *min_child_weight*. To ensure the reliability of our results, we employed a 5-fold cross-validation strategy. In 5-fold cross-validation, the dataset was randomly partitioned into five equally sized folds, where the model was trained on four of the folds and validated on the remaining fold iteratively. This approach provides a robust assessment of the model’s generalization performance across various data subsets (Zou et al., 2022). We employed feature importance analysis with Python to quantify the relative importance of input variables (age, performance results of Clean and Jerk, individual BW, and athlete’s belonging BW class) in our XGBoost models for identifying doping suspicions. This analysis allowed us to visualize the significance of each feature. To leverage the capabilities of machine learning, we employed a Multilayer Perceptron (MLP) model. This model is a type of artificial neural network that processes information in layers, aiming to predict outcomes based on patterns learned from data. For our implementation, we standardized the input data, ensuring it has a mean of 0 and a standard deviation of 1, which helps the model perform effectively across different types of data. The training of the MLP involved utilizing the standard backpropagation algorithm, which enables the model to learn from its mistakes during the training process, adjusting its internal parameters to improve accuracy (Taye, 2023). The MLP had 5 input units representing various features and 1 output unit to predict the binary outcome: 0 for “*not-sanctioned”* and 1 for “*sanctioned*.” To optimize the performance of the MLP model, we experimented with varying the number of neurons and the configuration of hidden layers. To systematically identify the best-performing model, we also implemented a grid search combined with 5-fold cross-validation.

## Results

### Weightlifting performance profiles and sanction trends across age groups and body weight classes in female weightlifting

**FIGURE 1:**
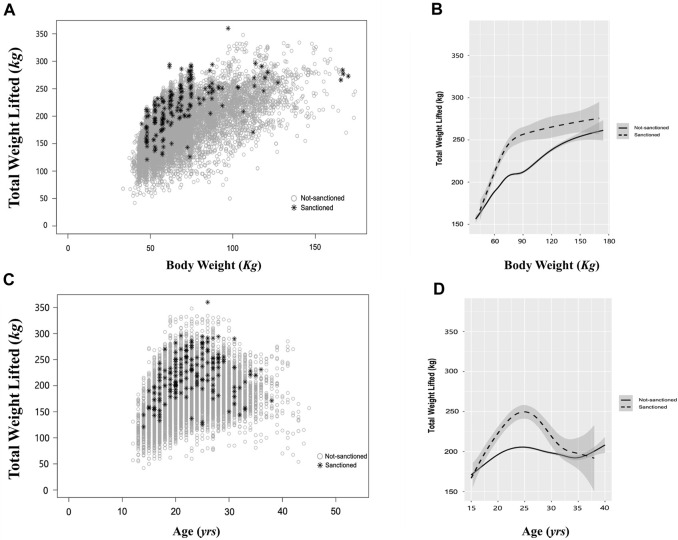
Performance Distribution and Trends in Female Weightlifters Across Age and Body Weight with Sanction Status Differentiation. **(A)** Scatter plot illustrating the distribution of performance across body weight in female weightlifters. **(B)** Line graph depicting the trend of performance across body weight. **(C)** Scatter plot displaying the distribution of performance across age in female weightlifters. Gray open circles represent not-sanctioned cases, and black star marks (*) represent sanctioned cases. **(D)** Line graph illustrating the trend of performance across age. *Gray open circles* denote not-sanctioned cases, while *black asterisks* indicate sanctioned cases in **(A, C)**. The solid line represents the mean performance of not-sanctioned cases, while the dashed line represents the mean performance of sanctioned cases in **(B, D)**.

The comprehensive analysis of female weightlifters’ performances, illustrated through the overall distribution and the average performance trend line across BWs (Figure 1A, B) and ages (Figure 1C, D) indicates that a performance trend of sanctioned cases consistently surpassed the average performance of the non-sanctioned counterparts. Of the 1,654 sanctioned cases, the distributions of sanctioned cases across age groups and BW classes were as follows: [Age groups: 14 cases in <15 group (0.8%), 505 cases in 15–19 group (30.5%), 571 cases in 20–24 group (34.5%), 388 cases in 25–29 group (23.5%), 148 cases in 30–34 group (8.9%), 26 cases in 35–30 group (1.7%), and 2 cases in ≥40 group (0.1%); BW groups: 219 cases in 49 kg (13.2%), 251 cases in 55 kg (15.2%), 226 cases in 59 kg (13.7%), 237 cases in 64 kg (14.3%), 427 cases in 76 kg (25.8%), 51 cases in 87 kg (3.1%), and 243 cases in +87 kg (14.7%) (Tables 1, 2). The results of the comparative analysis regarding weightlifting performance levels of the athletes and their sanction statuses across the age groups are represented in Table 1. Doping prevalence, calculated as sanctioned cases divided by the number of analyzed cases, in each age and BW class was as follows: [Age groups: 17.3% (*14/81*) in <15 group, 5.9% (*505/8,636*) in 15–19 group, 11.8% (*571/4,834*) in 20–24 group, 15.6% (*388/2,485*) in 25–29 group (23.5%), 18.09% (*148/818*) in 30–34 group (8.9%), 16.15% (*26/161*) in 35–30 group (1.7%), and 5.88% (*2/34*) in ≥40 group (0.1%); BW groups: 8.3% (*219/2,642*) in 49 kg, 9.9% (*251/2,539*) in 55 kg, 8.6% (*226/2,628*) in 59 kg, 8.9% (*237/2,678*) in 64 kg, 10.7% (*427/4,007*) in 76 kg, 6.3% (51/813) in 87 kg, and 13.9% (243/1751) in +87 kg] (Tables 1, 2). The results of the comparative analysis regarding weightlifting performance levels of the athletes and their sanction statuses across the BW classes are represented in Table 2. Sanctioned prevalence in each performance level, striated by age and BW class, was as follows: [*by age group*: 27.1% (*42/155*) in Top 1%, 27.3% (*186/681*) in Top 1%–5%, 20.1% (*165/823*) in Top 5%–10%, 12.3% (*305/2,490*) in Top 10%–25%, 9.9% (*415/4,174*) in Top 25%–50%, 8.2% (*344/4,219*) in Top 50%–75%, 5.7% (*150/2,613*) in Top 75%–90%, 2.5% (*47/1903*) in Top 90%–100%; *by BW class*: 38.1% (*59/155*) in Top 1%, 27.5% (*185/681*) in Top 1%–5%, 20.5% (*164/823*) in Top 5%–10%, 16.7% (*422/2,490*) in Top 10%–25%, 10.6% (*438/4,174*) in Top 25%–50%, 6.8% (*286/4,219*) in Top 50%–75%, 3.0% (*79/2,613*) in Top 75%–90%, 1.1% (*21/1903*) in Top 90%–100%]. In summary, cases of ADRVs were the highest in 20–24 age group; however, the prevalence was the highest in 30–34 age group. Thereafter, the prevalence was high in the order of 35–39, 25–29, 20–24, 15–19. Considering the relative small numbers of analyzed cases in <15 and ≥40 groups, doping prevalence appeared to increase as athletes age. Cases of ADRVs were the highest in 76 kg BW class; however, the prevalence was the highest in +87 kg BW class with no specific discernible pattern of doping prevalence across BW class. While there was no discernible pattern in the absolute number of ADRV cases across performance levels, the prevalence was highest in the Top 1%, gradually decreasing as performance levels decreased. These observations highlight the significance of considering demographic factors in assessing performance outcomes and potential doping suspicions in female weightlifting.

**TABLE 1:** Distribution of sanctioned cases across age groups and performance levels in female weightlifting analysis of 17,058 cases.

Age group	Top 1% (*n = 155*)	Top 1%–5% (*n = 681*)	Top 5%–10% (*n = 823*)	Top 10%–25% (*n = 2,490*)	Top 25%–50% (*n = 4,174*)	Top 50%–75% (*n = 4,219*)	Top 75%–90% (*n = 2,613*)	Top 90%–100% (*n = 1903*)	ADRVs
<15 (*n = 81*)	0	1	0	4	4	4	1	0	14 (*17.3%*)
15–19 (*n = 8,636*)	24	59	58	96	137	98	26	7	505 (*5.9%*)
20–24 (*n = 4,834*)	11	72	63	111	137	114	52	11	571 (*11*.*8%*)
25–29 (*n = 2,485*)	4	42	32	66	105	79	41	19	388 (*15*.*6%*)
30–34 (*n = 818*)	3	12	12	24	26	36	26	9	148 (*18*.*1%*)
35–39 (*n = 161*)	0	0	0	4	5	12	4	1	26 (*16*.*2%*)
≥40 (*n = 34*)	0	0	0	0	1	1	0	0	2 (*5*.*9%*)
ADRVs	42 (*27.1%*)	186 (*27.3%*)	165 (*20.1%*)	305 (*12.3%*)	415 (*9.9%*)	344 (*8.2%*)	150 (*5.7%*)	47 (*2.5%*)	1,654

*The percentages in the parentheses denote the number of ADRVs, per analyzed cases in the matched age group (rows) or in the matched performance level (columns).

**TABLE 2:** Distribution of sanctioned cases across body weight classes and performance levels in female weightlifting analysis of 17,058 cases.

BW class	Top 1% (*n = 155*)	Top 1%–5% (*n = 681*)	Top 5%–10% (*n = 823*)	Top 10%–25% (*n = 2,490*)	Top 25%–50% (*n = 4,174*)	Top 50%–75% (*n = 4,219*)	Top 75%–90% (*n = 2,613*)	Top 90%–100% (*n = 1903*)	ADRVs
49 (*n = 2,642*)	9	21	21	65	59	33	10	1	219 (*8*.*3%*)
55 (*n = 2,539*)	5	23	23	62	74	48	14	2	251 (*9*.*9%*)
59 (*n = 2,628*)	4	24	18	46	55	58	17	4	226 (*8*.*6%*)
64 (*n = 2,678*)	12	31	22	70	53	39	7	3	237 (*8*.*9%*)
76 (*n = 4,007*)	23	60	44	96	103	66	25	10	427 (*10*.*7%*)
87 (*n = 813*)	2	10	4	14	15	6	0	0	51 (*6*.*3%*)
87+ (*n = 1751*)	4	16	32	69	79	36	6	1	243 (*13*.*9%*)
ADRVs	59 (*38.1%*)	185 (*27.5%*)	164 (*20.5%*)	422 (*16.7%*)	438 (*10.6%*)	286 (*6.8%*)	79 (*3.0%*)	21 (*1.1%*)	1,654

*The percentages in the parentheses denote the number of ADRVs, per analyzed cases in the matched BW, class (rows) or in the matched performance level (columns).

### Performance of prognostic models for doping suspicions among female weightlifters

The performance of the prognostic models for doping suspicious in female weightlifting, including logistic regression, XGBoost model, MLP model, and the optimal Ensemble model, is detailed in Table 3. The logistic regression model demonstrated performance on the training set, achieving an accuracy of 0.710, AUC-ROC of 0.695, and F1 score of 0.300, while in the test dataset, it exhibited enhanced efficacy with an accuracy of 0.852, AUC-ROC of 0.761, and F1 score of 0.581. Our investigation of the performance of the XGBoost model, with varying tree depths ranging from 2 to 7, revealed that the highest achievement in the training dataset was observed in the model with a depth of 7, while the highest performance in the test dataset was attained by the model with a depth of 6. Specifically, during 5-fold cross-validation, the model with a depth of 7 exhibited an accuracy of 0.818, an AUC-ROC of 0.695, and an F1 score of 0.3207 within the training dataset; and the model with a depth of 6 exhibited an accuracy of 0.875, an AUC-ROC of 0.790, and an F1 score of 0.621 within the test dataset. Across all depths, BW emerged as the most important feature to the models’ prediction for the doping, with gains of 0.576, 0.539, 0.513, 0.552, 0.461 and 0.489 at depth from 2 to 7, *respectively*. Following BW, snatch record exhibited significant importance, particularly in the models with depths 3 to 5, serving as the second most important feature for the models’ prediction performance. However, as the depth increased to 6 and 7, BW class emerged as the second most important feature while snatch result became the third. Jerk record was not deemed as important, with gains less than 0.1 in models with depth from 3 to 7; it was not considered by the model with depth 2. Age appeared to be the least important feature as it was not considered by the models until depth increased to 6 and 7 with the gains less than 0.005 at these depths. Feature importance plots for our XGBoost models are shown in Supplementary Figure S1.

**TABLE 3:** Performance metrics of AI-Powered models for doping suspicion prediction in female weightlifting.

Model		Train (*5-fold CV*)			Test		
		Accuracy	AUC-ROC	F1 score	Accuracy	AUC-ROC	F1 score
Logistic Regression		0.710	0.695	0.300	0.852	0.761	0.581
XGBoost	Depth: 2	0.844	0.636	0.254	0.784	0.685	0.457
	Depth: 3	0.710	0.6623	0.285	0.796	0.730	0.471
	Depth: 4	0.773	0.677	0.305	0.796	0.746	0.471
	Depth: 5	0.815	0.689	0.308	0.830	0.777	0.546
	Depth: 6	0.798	0.694	0.315	0.875	0.790	0.621
	Depth: 7	0.818	0.695	0.321	0.864	0.780	0.600
MLP	Hidden: 2	0.764	0.701	0.305	0.830	0.748	0.546
	Hidden: 3	0.772	0.705	0.311	0.875	0.760	0.621
	Hidden: 4	0.716	0.709	0.315	0.886	0.766	0.615
Ensemble	Depth: 5 Hidden: 4	0.693	0.697	0.313	0.875	0.783	0.645

CV, cross validation; MLP, multilayer perceptron; AUC-ROC, area under the curve of receiver operating characteristics.

In our investigation of the Multilayer Perceptron (MLP) model’s performance with varying number of hidden units from 2 to 4, the model with 3 hidden units demonstrated highest performance in both the training and test datasets. Employing a 5-fold cross-validation framework, this model exhibited an accuracy of 0.772, an AUC-ROC of 0.705, and an F1 score of 0.311 in the training dataset, while in the test dataset, it exhibited enhanced efficacy with an accuracy of 0.875, an AUC-ROC of 0.760, and an elevated F1 score of 0.621. Due to the ‘black-box’ nature MLP models, we were not able to quantify the relative importance of the input variables.

While the XGBoost model with a tree depth of 6 and the MLP model with 3 hidden units individually demonstrated the best performance, our investigation extended to the exploration of ensemble models to further improve predictive capabilities. Optimal result was attained in the Ensemble model combining XGBoost with a tree depth of 5 and MLP with 4 hidden units. This combination yielded notable results, achieving an accuracy of 0.875, an AUC-ROC of 0.783, and an F1 score of 0.645 in the test dataset. This integration of machine learning models demonstrated a synergistic effect, augmenting predictive capacity for the identification of doping suspicions in female weightlifting. Among female weightlifters participated in 2008 Beijing Olympics, 2012 London Olympics, and 2016 Rio de Janeiro Olympics, the model correctly identified 10, 9, and 2 athletes for potential doping suspicions out of 15, 16, and 8 athletes who were subsequently sanctioned, *respectively*, achieving a prediction rate of 66.7%, 56.3%, and 25%, *respectively*, while erroneously identifying 6, 13, and 4 athletes for ADRV out of 73, 74, and 83 athletes who were not sanctioned (Table 4).

**TABLE 4:** Ensemble model performance in predicting doping suspicions among female weightlifters–2008, 2012, 2016 olympics.

Best prediction by ensemble		References	
		Not -sanctioned	Sanctioned
2008 Beijing	Not-Sanctioned	67	5
	Sanctioned	6	10
2012 London	Not-Sanctioned	61	7
	Sanctioned	13	9
2016 Rio de Janeiro	Not-Sanctioned	79	6
	Sanctioned	4	2

## Discussion

In this study, we demonstrated the efficacy of APP in predicting doping suspicion among elite female weightlifters through our developed Ensemble Model, leveraging the advantages of XGBoost and MLP. Cognizant of prior attempts to utilize the APP in anti-doping practices, our study introduces a substantive advancement in methodology. In contrast to earlier investigations employing the Bayesian spline model or the delta excess performance model, which primarily focus on analyzing standardized performances and measuring unusual improvements through yearly changes, our distinguished approach enables the classification of athletes into ‘sanctioned’ or ‘not-sanctioned’ categories, offering a proactive strategy in doping suspicion identification by incorporating artificial intelligence algorithms. Our Ensemble model, strategically combining the strengths of both XGBoost and MLP, utilized pooled data including ages, BWs, performance results and sanction status of elite female weightlifters; it adeptly identified doping suspicions among weightlifters participated in 2008 Beijing Olympics, 2012 London Olympics, and 2016 Rio de Janeiro Olympics. These findings highlight the pragmatic applicability of APP in doping suspicion prediction and demonstrate its potential for practical deployment in targeted doping control efforts, efficiently identifying athletes with high suspicion levels.

While ADRV instances are dispersed across diverse age groups, our comparative analysis suggests a potential inclination towards doping in athletes approaching the age of peak performance. It has been previously reported that elite female weightlifting performances exhibit a peak around the median age of 25.6, succeeded by a decrement with advancing age (Huebner et al., 2021). Our result concurs with this trend, revealing that female weightlifters aged 24 (20–24 age group) performed optimally (Figure 1B, D). Remarkably, within this age cohort, and particularly within the top 25%–50% performance level category, we identified the highest incidence of sanctioned cases. A parallel study analyzing APP in elite male weightlifters also yielded analogous outcomes (Ryoo et al., 2022), further accentuating a potential doping proclivity among female weightlifters in their early twenties. The highest propensity in this age group may be conceivably driven by the substantial pressure to attain peak performance during this phase or to forestall the performance decline concomitant with aging (Petróczi and Aidman, 2008; Huebner and Perperoglou, 2019). Our analysis substantiates the underlying hypothesis by revealing significantly inflated average performance results (*kg*) among sanctioned athletes compared to their non-sanctioned counterparts across age groups and BW classes (Figure 1B, D). Considering these observations, APP emerges as a valuable source, enabling the establishment of predictive performance ranges for female weightlifters in distinct age groups and BW classes. Furthermore, the utility of APP extends to the identification of abnormal deviations indicative of potential doping practices, thereby reinforcing its value in the context of anti-doping strategies.

The incidence of ADRVs varied from 0.96% to 2.45% during the period spanning 1987 to 2013 (de Hon et al., 2015); this fluctuation could be attributed to the inherent challenges associated with ambiguous criteria and subjectivity in the identification of positive cases (Nissen-Meyer et al., 2022). Additionally, limitations in the ABP monitoring system and the inefficiency of current athlete selection methods, which rely on finishing position, randomization, or specific targeting for doping tests, may contribute to these fluctuating rates (Maennig, 2014). Recognizing these challenges, our proposed solution, the implementation of an APP-based prognostic model, has the potential to significantly enhance the efficiency of doping tests by systematically identifying and suggesting potential doping suspicions. The application of mathematical representations to performance data has found utility in sports in the identification of doping suspicions (Jones and Vanhatalo, 2017). One such example is the utilization of the concept of critical power (CP), originally describing the hyperbolic relationship between power output and the time it can be sustained. This concept has been applied in various timed sports where the velocity of athletes or teams is available, allowing for performance prediction. Models based on CP have been proposed as a valuable approach in anti-doping practice as they have demonstrated efficacy in describing mean-maximal power profiles collected from athletes or teams during competitions and detecting performances that exceed typical errors (Puchowicz et al., 2018). The parameters derived from the power-time relationship in sports, while valuable, are conventionally limited to performance during constant power output exercise. Consequently, models based on CP cannot be universally implemented across diverse sports (Jones and Vanhatalo, 2017). To address the limitation of CP-based models, the integration of AI holds promise, as AI-powered algorithms can excel in analyzing extensive datasets, offering a more comprehensive and refined evaluation of athletes’ profiles for the application for the identification of doping suspicions in sports (Chmait and Westerbeek, 2021; Molavian et al., 2023). Through the detection of irregular patterns, trends, and anomalies, AI systems can support stakeholders in pinpointing athletes engaged in prohibited practices, thus serving as an independent and valuable criterion for selecting individuals for doping tests.

The utilization of XGBoost, a gradient-boosted decision trees (GBDT) algorithm, XGBoost, presents several advantages in developing the classifier model for identifying doping suspicions among elite female weightlifters. GBDT is well known for its ability to handle complex, non-linear relationships among input variables and the target outcome, making it particularly effective in capturing subtle patterns in diverse datasets (Friedman, 2001). By incorporating multiple input variables, including age, performance record of clean and jerk, individual body weight, and athlete’s belonging body weight class, our model could leverage the collective information provided by these variables to enhance predictive accuracy. GBDT inherently performs feature selection during model training, automatically identifying the most informative variables for predicting the target outcome (Upadhyay et al., 2021). This capability ensured that our model focused on relevant input features, optimizing its predictive performance. Our feature importance analysis revealed that body weight emerged as the most important feature across all depths, followed by the snatch record and body weight class, highlighting their crucial roles in identifying doping suspicions. This capability ensured that our model focused on relevant input features, optimizing its predictive performance. Traditional machine learning methods, such as GBDT, have long been recognized as dominant in tabular data modeling, exhibiting superior performance over deep learning (Chen and Guestrin, 2016; Shwartz-Ziv and Armon, 2022). However, recent efforts have been made to apply deep learning networks to tabular data, with some neural network models claimed to outperform GBDT (Borisov et al., 2022). Consequently, experts in relevant fields suggest implementing hybrid methods to leverage the flexibility of neural networks while retaining the inductive biases of GBDT (Borisov et al., 2022). In consideration of these, we aimed to enhance predictive by adopting a hybrid approach through developing an Ensemble model. Our prediction models exhibited relatively good performance, with the Ensemble model being as the best performing model, achieving the highest F1 score. Considering that F1 score is a fundamental metric for evaluating the effectiveness and the performance of the classification models, describing the harmonic mean of the quality of positive predictions (precision) and the sensitivity of correct detections of positive events (recall) (Sokolova and Lapalme, 2009), the Ensemble model appeared to outperform logistic regression, XGBoost and MLP models. The Ensemble model demonstrated prediction rates of 66.7%, 56.25%, 25% for ADRV in 2008 Beijing Olympics, 2012 London Olympics, and 2016 Rio de Janeiro Olympics, *respectively*. These indicate the significance of our effort to implement a hybrid method for enhancing predictability of identification of doping.

While athletes can exhibit exceptional performance improvements beyond typical ranges through doping, others may achieve substantial performance enhancements through training. Consequently, criticism has been directed towards selecting athletes for doping tests solely based on performance results (de Hon et al., 2015). To address this concern, the ongoing development of the APP system, incorporating higher qualitative and quantitative data to enhance its predictive capabilities for identifying doping suspicions. Acknowledging the observed occurrence of ADRVs across all age groups, BW classes, and performance levels, the incorporation of demographical and individualized performance changes over time into the APP system may enhance its utility in anti-doping strategies. The implication of data/AI-driven approach in identification of doping suspicions, akin our Ensemble model, demonstrated its proactive and efficient practicality, and it may substantially enhance the efficiency and objectivity in the selections for doping tests when implemented in diverse sports, particularly with the integration of more advanced APP system.

A limitation of our study is the constrained set of available APPs (age, discrete performance outcomes, individual BW and belonging BW class) utilized in developing the prediction models, primarily owing to restricted public availability. This limitation may potentially impact the robustness and practical applicability of the models. For future studies in anti-doping research, the development of prediction models tailored to specific sports, utilizing APP and demographic features unique to each discipline, is recommended. Establishing easily accessible and standardized systems for managing APP within each sport would be essential for collecting consistent and relevant data, enabling the construction of accurate and efficient prediction models. By focusing on sport-specific systems, researchers can ensure that prediction models are optimized for the intricacies of each athletic discipline, thereby enhancing the effectiveness of anti-doping efforts.

In conclusion, we have effectively demonstrated the efficacy of APP in predicting doping suspicions through the implementation of an AI-powered prognostic model among elite female weightlifters. Addressing the existing inefficiencies in current doping test selection criteria marked by ambiguity and subjectivity, the constructive deployment of APP-based prediction models is advocated. This methodology represents a pioneering initiative, introducing a novel approach to augment the efficacy of doping tests.

## Data Availability

The raw data supporting the conclusion of this article will be made available by the authors, without undue reservation.
